# Successful use of venous graft from native liver with hepatocellular carcinoma during living donor liver transplantation with no impact on recurrence rate: A retrospective cohort study

**DOI:** 10.1016/j.amsu.2022.104714

**Published:** 2022-09-15

**Authors:** Hazem Mohamed Zakaria, Emad Hamdy Gad, Nahal Kamel Gaballa, Ahmed Nabil Sallam, Islam Ismail Ayoub, Mohamed Eltabbakh, Shimaa Saad Elkholy, Sameh Abokoura, Taha Yassein, Osama Hegazy, Hany Abdelmeguid Shoreem, Hossam Eldeen Mohamed Soliman, Amr Ahmed Aziz, Mohammad Taha

**Affiliations:** a -Department of Hepatopancreatobiliary and Liver Transplant Surgery, National Liver Institute, Menoufia University, Menoufia, Egypt; b -Department of Anesthesia and Intensive Care, National Liver Institute, Menoufia University, Menoufia, Egypt; c -Department of Hepatology and Gastroenterology, National Liver Institute, Menoufia University, Menoufia, Egypt; d -Department of Pathology, National Liver Institute, Menoufia University, Menoufia, Egypt; e -Department of Diagnostic and Intervention Radiology, National Liver Institute, Menoufia University, Menoufia, Egypt

**Keywords:** Liver transplantation, Hepatocellular carcinoma, Alfa-fetoprotein, Recurrence

## Abstract

**Introduction:**

There are still debates regarding using portal vein (PV) from liver with hepatocellular carcinoma (HCC) for vascular reconstruction. This study aimed to assess the feasibility and patency of PV venous graft from an explanted liver with HCC for the reconstruction of the hepatic veins tributaries or PV in living donor liver transplantation (LDLT) and to see if it has any risk on recurrence of HCC.

**Patient and methods:**

We conducted a retrospective study on 81 patients with HCC who underwent LDLT from April 2004 to July 2022.

**Results:**

Venous graft from native liver PV was used for vascular reconstruction in 31 patients as follows; reconstruction of V5 in 7 patients, V8 in 4 patients, V6 in 3 patients, combined V5 and V8 in 4 patients, V6 with V5/V8 in 5 patients, and as Y shape venous graft for 2 PV reconstruction in 8 patients. The implantation of the new conduit PV graft after reconstruction of the anterior sector tributaries was direct to the IVC in 8 patients, and to the common orifice of the left and middle hepatic veins in 12 patients. The 1 month, 3 months, and 1-year overall patency of the venous graft was 93.5%, 90.3%, and 84%, respectively. Nine patients had recurrent HCC. In multivariate analysis, the independent risk factors for HCC recurrence were AFP >400 ng/mL (HR = 1.47, 95% CI: 1.69–2.31, P = 0.01), moderate/poor differentiated tumor (HR = 3.06, 95% CI: 2.58–6.29, P = 0.02), and microvascular invasion (HR = 2.51, 95% CI: 1.05–1.93, P = 0.01). Using a PV venous graft had no risk factor for HCC recurrence (P = 0.9).

**Conclusion:**

The use of PV venous graft of native liver with HCC for venous reconstruction is a feasible and valuable option in LDLT with good patency rates and no risk of HCC recurrence.

## Highlights

• The use of PV venous graft of native liver with HCC for venous reconstruction is a feasible and valuable option in LDLT.
• It has good patency rates and no risk on HCC recurrence.
• The independent risk factors for HCC recurrence in our study were AFP >400 ng/mL, moderate/poor differentiated tumor, and microvascular invasion.

## 1 Introduction

Hepatocellular carcinoma (HCC) is one of the main indications for liver transplantation (LT). However there are many risk factors for tumor recurrence which necessitate strict preoperative assessment and selection of the patient candidate for transplant [[1], [2], [3]].

Living donor liver transplantation (LDLT) is a safe source for liver allografts as deceased organ donation remains scarce. In LDLT, vascular grafts are frequently necessary because of anatomical variations in donors like trifurcated portal vein, short length of donor graft vessels, and recipient portal vein thrombosis [3,4].

The right lobe is commonly used to provide adequate volume in an adult recipient. The middle hepatic vein (MHV) tributaries must be reconstructed during the back table procedure. In the absence of adequate graft venous drainage, the portal inflow can cause damaging effects on the liver allograft with postoperative liver dysfunction and small-for-size syndrome [4,5].

The commonly used vascular grafts for reconstruction are the left portal vein, recanalized umbilical vein, hepatic veins, ovarian vein, recipient saphenous vein, cryopreserved vessels, or expanded polytetrafluoroethylene (PTFE) synthetic grafts. Various centers have successfully reported their experience using natural or synthetic grafts with a variable incidence of patency and recipient outcomes [[5], [6], [7]].

The recipient portal vein may be unsuitable for reconstruction in cases with portal vein thrombosis (PVT) or HCC, especially if the tumor was near to the main PV [7,8]. The purpose of this study was to evaluate the feasibility and patency of portal vein venous graft from explanted liver with HCC to reconstruct the hepatic veins tributaries or PV of the liver graft and to see if it has any risk on recurrence of HCC after LDLT.

### 1.1 Patients and methods

We conducted a retrospective cohort study on 81 patients with HCC who underwent living donor LT from April 2004 to July 2021, at National Liver Institute, Menoufia University, Egypt. After receiving approval from the ethics committee and our institutional review board, the data were collected from the transplant database and patients' medical records in accordance with the roles outlined in the 1975 Helsinki Declaration. All patients signed a consent form to allow their data to be used in research. The research was registered with the Chinese clinical trial registry, the identification number is ChiCTR2000030403. The work has been matched in line with the Strengthening the Reporting of Cohort Studies in Surgery (STROCSS) criteria [9].

Patients with first month mortality or lost follow up were excluded from our study for accurate detection of HCC recurrence. All collected data were studied and analyzed statistically for its risk with the recurrence of HCC like preoperative demographic and clinical data, operative data (graft recipient weight ratio, cold ischemia time (CIT), warm ischemia time (WIT), total operative time (TOT), amount of blood transfusion), postoperative data and pathological study of explanted liver.

All patients underwent a recent multislice triphasic computed tomography (CT) scan or magnetic resonance image (MRI) for diagnosis of HCC within 1month before liver transplant. Cases with HCC underwent metastatic workup such as positron image tomography (PET) to exclude extrahepatic metastasis or vascular invasion. The selection criteria for LDLT in patients with HCC were categorized as within Milan criteria, beyond Milan criteria, within University of California San Francisco (UCSF) criteria, and beyond UCSF criteria [10].

### 1.2 Explanted portal vein harvest

After recipient hepatectomy, the portal vein of the explanted liver was harvested on the back table. Our role in taking the portal vein graft was that the tumor should be at least 3 cm away from the hilar or first division portal vein. The extrahepatic portion and a good length of intrahepatic portal vein were carefully dissected by ligation of the connected small branches. We used mainly the main PV with the left PV, if the harvested segment of PV was short, an extra part of recanalized umbilical vein or parts of explanted liver hepatic veins were used. The segment that was harvested from the portal vein was preserved in heparinized saline until the time of anastomosis to the graft veins. The harvested segment of the portal vein was used for reconstruction of the middle hepatic vein tributaries (V5, V8), right inferior hepatic vein, or graft with 2portal veins by proline 6/0.

The postoperative immunosuppressant regimen was methylprednisolone, mycophenolate mofetil, and calcineurin inhibitors (CNI). After 3 months, we tried to shift CNI to other immunosuppressants as mammalian target of rapamycin (m-TOR) inhibitors like Everolimus/Sirolimus or in combination with a small dose of CNI due to its risk on recurrence of HCC.

The protocol of follow up of the graft post-transplant was by Doppler ultrasound every day until patient discharge then weekly or every 2 weeks in the first 3 months according to the follow up visit at the outpatient clinic, then monthly during the first year. The CT abdomen and serial Alfa-fetoprotein serum level were every 3–6 months in the first 2 years, then yearly. The duration of follow up was at least 6 months from the last recipient, with a mean duration of follow up 63.5 months (range 6–198 months).

## 2 Statistical analysis

Statistical analysis was performed using the SPSS program version 23.0 (SPSS Inc., Chicago, IL, USA). Continuous variables were shown as mean, range and were compared using the Mann Whitney *U* test in non-parametric data or by using the student-t test. Categorical or discrete variables were expressed as frequency or proportions and were compared using the Chi-square test or Fisher's exact test. The Kaplan-Meier curve was used for analysis of overall survival and tumor free survival. A probability value < 0.05 was considered statistically significant for all tests.

## 3 Results

Eighty-one patients who underwent LDLT for HCC were included in our study. Their demographic, operative, and postoperative data are shown in Table 1, Table 2. Nine patients had recurrent HCC after LT, their mean age was 54.4 years, and all were male. The main cause of HCC was the hepatitis C virus (HCV) (88.9%). Five patients (55.6%) with recurrent HCC had preoperative multiple bilateral lesions, and 4 patients (44.4%) had only 1 HCC in the right lobe of the liver. Preoperative serum AFP levels were >400 ng/ml in 4 patients (44.4%) with HCC recurrence.

**Table 1:** Preoperative data of HCC cases.

	HCC without recurrence (n = 72)	HCC with recurrence(n = 9)	*P*- value
Age (year)			0.02
-Mean± SD	48.7 ± 6.7	54.4 ± 5.5	
-Range	(33–66)	(47–60)	
**Gender**			1.0
-Male	68(94.4%)	9(100%)	
-female	4(5.6%)	0	
**Viral markers**			0.89
-HCV	67(93%)	8(88.9%)	
-HBV	3(4.2%)	0	
-others	2(2.8%)	1(11.1%)	
**Child score**			0.36
-A	10(13.9%)	3(28.6%)	
-B	33(45.8%)	3(28.6%)	
-C	29(40.2%)	3(42.8%)	
**MELD score**			
-Mean± SD	14 ± 4	14 ± 4	
-Range	(7–24)	(8–19)	
MELD≤10	11(15.3%)	2(22.2%)	0.741
MELD>10	61(84.7%)	7(77.8%)	0.75
**Ascites**			0.70
-no	15(20.8%)	3(33.3%)	
-mild	14(19.4%)	1(11.1%)	
-moderate	36(50%)	5(55.6%)	
-marked	7(9.7%)	0	
**AFP (ng/mL)** (normal <12 ng/mL)			0.01
Mean ± SD	118 ± 505	736 ± 1127	
Range	(0.5–4022)	(3.8–3041)	
≤400	67(93.1%)	5(55.6%)	
>400	5 (6.9%)	4(44.4%)	
**Bilirubin (mg/dl)**			0.4
-Mean± SD	3.1 ± 3.2	2.6 ± 1.4	
-Range	(0.5–21.9)	(0.8–5.3)	
**Albumin (g/dl)**			
-Mean± SD	2.7 ± 0.6	3.1 ± 0.8	0.2
-Range	(2–4)	(2–4)	
**INR**			0.9
-Mean± SD	1.5 ± 0.3	1.5 ± 0.4	
-Range	(1–2.6)	(1–2.3)	
**ALT (U/L)**			0,05
-Mean± SD	61 ± 48	42 ± 21	
-Range	(10–226)	(16–69)	
**Neutrophil/lymphocyte**			0.03
<4	59(81.9%)	5(55.6%)	
≥4	13(18.1%)	4(44.4%)	
**PVT**	11(15.3%)	2(22.2%)	0.63
**Ablative therapy**			0.73
**-**yes	28(39.9%)	4(44.4%)	
-no	44(61.1%)	5(55.6%)	

HCC (hepatocellular carcinoma), SD (standard deviation), HCV (hepatitis C virus), HBV (hepatitis B virus), MELD (model of end-stage liver disease), SD (standard deviation), AFP (alfa-fetoprotein), ng (nanogram), mL (milli Leter), INR (international normalized ratio), ALT (Alanine aminotransferase), PVT (portal vein thrombosis).

**Table 2:** Operative, postoperative and pathological data of HCC cases.

	HCC without recurrence(n = 72)	HCC with recurrence(n = 9)	*P*- value
**Type of graft**			0.91
Right lobe	69(95.8%)	9(100%)	
Left lobe	3(4.2%)	0	
**Actual graft weight (gm)**			0.98
Mean ± SD	860 ± 157	861 ± 122	
Range	(450–1200)	(650–1000)	
**GRWR**			0.15
Mean ± SD	1 ± 0.2	0.97 ± 0.13	
Range	(0.7–1.6)	(0.8–1.2)	
**CIT (minutes)**			0.18
Mean ± SD	61 ± 25	77 ± 32	
Range	(20–126)	(40–120)	
**WIT (minutes)**			0,21
Mean ± SD	52 ± 16	59 ± 14	
Range	(30–105)	(45–90)	
**Operative time (hour)**			0.26
Mean ± SD	14.4 ± 2	15.2 ± 1.8	
Range	(8–18)	(12.5–19)	
**Blood transfusion (unit)**			0,20
Mean ± SD	5 ± 6	3 ± 3	
Range	(0–21)	(0–10)	
**Plasma transfusion (unit)**			0.76
Mean ± SD	6 ± 9	5 ± 5	
Range	(0–30)	(0–14)	
**Portal vein venous graft**	28(38.9%)	3(33.3%)	0.9
**Tumors number**			0.89
single	35(48.6%)	4(44.4%)	
multiple	37(51.4%)	5(55.6%)	
**Tumor site**			0.25
Right	35(48.6%)		
Left	13(18.1%)	4(44.4%)	
bilateral	24(33.3%)	5(55.6%)	
**Largest tumor diameter (cm)**			0.54
-Mean± SD	2.7 ± 1.1	2.9 ± 1.1	
-Range	(1–5)	(2–5)	
**Tumor differentiation**			0.01
Well	21/65(32.3%)	0	
Moderate/poor	44/65(67.7%)	9(100%)	
**Pathological Tumor grade**			**0.03**
I,II	39/65(60%)	3(33.3%)	
III,IV	26/65(40%)	6(66.7%)	
**Capsule**			0.83
Present	10(13.9%)	2(22.2%)	
Absent	62(86.1%)	7(77.8%)	
**Microvascular invasion**	17(23.6%)	5(66.7%)	**0.01**
**Milan criteria**			0.44
Within	52(72.2%)	5(55.6%)	
Beyond	20(27.8%)	4(44.4%)	
**UCSF criteria**			0.37
within	59(81.9%)	6(66.7%)	
Beyond	13(18.1%)	3(33.3%)	
**Immunosuppressant**			0.06
CNI	49(68.1%)	4(44.4%)	
m-TOR	23(31.9%)	5(55.6%)	
**Hospital stay (days)**			0.33
Mean ± SD	22 ± 9	26 ± 6	
Range	(14–51)	(17–35)	

HCC (hepatocellular carcinoma), CIT (cold ischemia time), WIT (warm ischemia time), SD (standard deviation), UCSF (University of California San Francisco criteria). CNI (calcineurin inhibitor), m-TOR (mammalian target of rapamycin inhibitor).

**Fig. 1:**
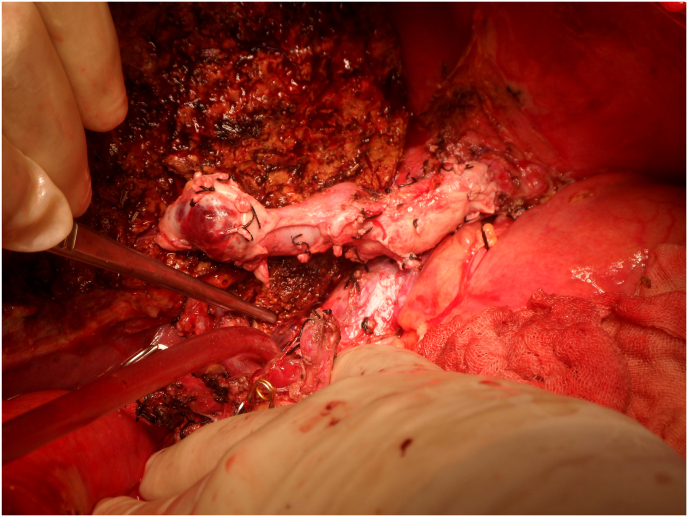
V5 reconstruction by PV graft.

**Fig. 2:**
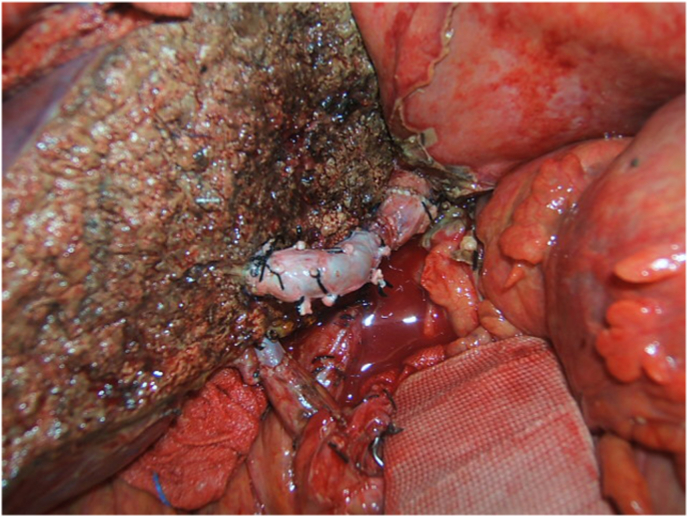
V8 reconstruction by PV graft.

**Fig. 3:**
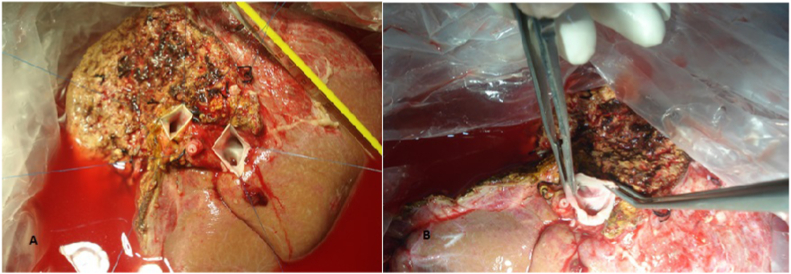
Y shaped venous graft reconstruction of 2 PV.

Venous graft from native liver portal vein was used for vascular reconstruction in 31 patients as follow; reconstruction of V5 in 7 patients (Fig. 1), V8 in 4 patients (Fig. 2), V6 in 3 patients, combined V5 and V8 in 4 patients, V6 with V5/V8 in 5 patients, and as Y shape venous graft for 2 PV reconstruction in 8 patients (Fig. 3). The mean length of the conduit was 6 cm (range 2–11 cm) and its mean diameter was 10 mm (range 7–15 mm). After reconstruction of the anterior sector tributaries (V5 and/or V8) the implantation of the new conduit portal vein graft direct to the IVC was done in 8/20 patients (40%), and to the common orifice of left hepatic vein and middle hepatic vein in 12/20 patients (60%). The 1 month, 3 month, and 1 year overall patency of the venous graft was 93.5%, 90.3% and 84% respectively. According to the technique of venous graft implant the 1month and 3 months patency was 87.5%, and 87.5% respectively in direct IVC implant versus 91.7% and 83.3% respectively, in common orifice implant with no significant difference. The 1year and 3 year liver graft survival rates were 88.2% and 79.6% respectively, with no risk of patency of the PV conduit on graft survival (P = 0.92).

The recurrence of HCC was mainly in the first 2 years post transplant, with a mean of 18.3 months and a range (11–28 months). Four patients (44.4%) had intrahepatic and extrahepatic recurrence, three patients (33.3%) had multiple hepatic recurrences, and two patients (22.3%) had only extrahepatic recurrence in the bones. Four patients (57.15%) with recurrent HCC were beyond Milan criteria, and three of them were also beyond UCSF criteria after studying the pathology of the explanted liver **(**Table 2**).** All patients with HCC recurrence had moderate/poor differentiated HCC, and five patients (66.7%) had microvascular invasion **(**Table 2**).**

The management was as follow: 3 patients underwent surgical excision of the recurrent HCC, 2 patients with bone metastasis underwent radiotherapy, and 4 patients underwent medical treatment with Sorafenib.

The risk factors for HCC recurrence in univariate analysis were; AFP >400 ng/mL (*P* = 0.01), neutrophil/lymphocyte ratio ≥4 (*P* = 0.03), moderate/poor differentiated tumor (*P* = 0.01), pathological tumor grades III and IV (*P* = 0.03), and microvascular invasion (*P* = 0.01). Using a venous graft from native liver for vascular reconstruction had no risk effect for HCC recurrence post LT (P = 0.9). In multivariate analysis the independent risk factors for HCC recurrence were AFP >400 ng/mL (HR = 1.47, 95% CI: 1.69–2.31, P = 0.01), moderate/poor differentiated tumor (HR = 3.06, 95% CI: 2.58–6.29, P = 0.02), and microvascular invasion (HR = 2.51, 95% CI: 1.05–1.93, P = 0.01).

## 4 Discussion

The need for vascular conduit became an important matter in LDLT due to the increased use of right lobe grafts and frequently encountered portal and hepatic vein anatomical variations [8,10,11]. The reconstruction of MHV tributaries has various techniques, including the use of a venous graft from the inferior mesenteric vein, great saphenous vein, femoral vein, internal jugular vein, recanalized recipient umbilical vein, cryopreserved vein grafts, or prosthetic grafts [8,11,12]. Most of these venous grafts have a small caliber or their procurement is hazardous to the recipient [13]. Prosthetic graft has a high liability for graft infection. Cryopreserved cadaveric graft although it is an attractive option but it is not available in some institutions with living donor programs and still a high possibility of transmission of uncommon pathogens and some studies showed lower long term patency rates than autologous vessel grafts [8,14,15].

Using vascular conduit in LDLT from a native liver with a tumor is still a debatable issue with multiple aspects that need discussion. The portal vein usually has the advantages of larger caliber, natural curvature along its course, and a thick wall [8]. Shi et al. [16], in their study on 113 patients with single HCC showed that in cases with HCC < 3 cm, the incidence of proximal micro metastasis is 0% if we had safety margin more than 2 cm, and 5.1% if the tumor >3 cm with same safety margin. They reported that 1 cm margin is safe in hepatic resection of HCC. So some authors thought that if the tumor was away from the hilar PV by > 2 cm, it was appropriate for harvesting the PV and using it as a venous graft, without any increased risk on recurrence of HCC [8,17], as we proposed in the selection of the PV graft in our study. Tashiro et al. [17], also showed successful use of an explanted right hepatic vein in vascular reconstruction as it is usually has a large diameter and intact intima, so it can eliminate additional hazardous surgery to the donor or the recipient in harvesting a suitable vascular graft, and it is accepted in cases with HCC if the tumor was more than 2 cm away from the right hepatic vein, as in the previous study with portal vein conduit.

In contrast, Kakodkar et al., showed successful use of cryopreserved portal vein, middle hepatic vein and recanalized umbilical vein from 6 explanted native livers who had no malignancy or thrombosis in other blood group matched recipients with unsuitable veins. They used these cryopreserved vascular grafts only after the exclusion of any intrahepatic malignancy in histopathological study of the explanted liver [7,11]. Different studies showed that the type of conduit had no impact on the patency of the reconstructed veins [6,18,19]. Borle et al., reported in their prospective observational study from 2014 to October 2015 on 88 patients with new MHV reconstruction that their first choice in conduit is PV graft (67/88, 86.7%), but in cases with PVT or HCC they prefer PTFE [20].

Some studies showed that the rate of thrombosis of the venous graft was 10–17% as seen in our study. Hwang et al., showed that if occlusion of the venous graft occurred after 2 weeks of transplant, the liver function and the survival rates would not be affected by the occlusion as seen in our study [21]. It has been reported that the liver graft may tolerate the accessory hepatic veins obstruction after 7–14 days after transplantation by the development of interlobar collaterals [20,22].

The cause of early venous graft occlusion may be due to technical issues or external compression with haematoma [8]. Shin et al. [23], reported clinically successful stenting of interposition grafts after acute obstruction (9/11 patients, 82%) and 63.6% of stent placements were in first 24 h after transplant. The mechanisms of late venous graft occlusions are different; it may be related to graft rotation, or decrease of inflow after regeneration or immunological factors after transplant [24,25]. Borle et al., showed that good monitoring of fluid balance in the early postoperative period by preventing persistently elevated central venous pressure to avoid any weak flow in the new veins also meticulously limited correction of mild coagulopathy in the perioperative period unless indicated [20].

In the previous series, they used explanted PV-umbilical vein complex from the recipient native liver in 66 patients with a patency rate of 89.4% at 1 month, and no difference from PTFE graft patency of 90%, also the technique of implantation direct to the IVC or as a common orifice with the right hepatic vein did not affect the patency rate of the venous graft as seen in our study [20]. Ikegami et al. also had the same findings that the type of graft implant did not affect its patency [8]. In one study by the Asan group, they used first a fence graft of cryopreserved iliac veins between the MHV tributaries and PTFE grafts; they supposed that it prevents the intimal proliferation that may occlude the PTFE graft [20].

A certain degree of graft congestion may be inevitable in the right lobe graft in LDLT, with subsequent inflammatory reaction and rabid regeneration of the non-congested segment. It has been suggested that graft congestion is a risk factor for HCC recurrence. Suh et al., reported that graft congestion >10%, microvascular invasion, and alpha-fetoprotein level >200 IU/L, were significantly risk factors for tumor recurrence [26,27], as seen in our study, but we did not investigate the effect and degree of graft congestion.

Halazum et al., developed a Model of Recurrence after Liver Transplant (MORAL) score in their prospective cohort study on 339 patient with HCC for the prediction of recurrence free survival after liver transplantation. In the pre-MORAL, the 3 preoperative independent predictors of worse survival were NLR ≥5, AFP >200, and tumor size >3 cm. In post MORAL, the postoperative independent predictors of survival were grade IV HCC, vascular invasion, tumor number >3, and size > 3 cm. They showed that the combined scores produce a combo-MORAL score that is a simple and highly accurate tool for the prediction of HCC recurrence [28]. Other studies also showed that NLR ≥4 was a strong independent risk factor for post LDLT tumor recurrence [29,30], in our study, NLR was a risk factor for HCC recurrence in univariate analysis.

The limitations of this study are the relatively small number of patients who used venous grafts, and the low incidence of recurrent HCC that may impact statistical bias. We did not measure the regeneration power of the anterior sector with and without occluded venous graft which needs another study. The percent of graft congestion that can affect graft function and its risk on HCC recurrence still needs further study.

**In conclusion**, the use of PV venous graft of native liver with HCC for venous reconstruction is a feasible and valuable option in LDLT, especially if other types of vascular grafts are not available, with good patency rates and no risk on HCC recurrence. The independent risk factors for HCC recurrence in our study were AFP >400 ng/mL, moderate/poor differentiated tumor, and microvascular invasion, that should be considered in selecting patients with HCC for liver transplantation, and also need close follow up for early detection of any tumor recurrence.

## Ethical approval

The research was conducted ethically in accordance with the World Medical Association Declaration of Helsinki. The study protocol was approved by the National Liver Institute committee and review board, Menoufia University, Egypt (**NLI: 5317**).

## Sources of funding

No funding.

## Author contribution

Hazem Mohamed Zakaria^1^, Emad Hamdy Gad^1^, Nahal Kamel Gaballa^2^, Ahmed Nabil Sallam^1^, Islam Ismail Ayoub^1^, Mohamed Eltabbakh^3^, Shimaa Saad Elkholy^4^, Sameh Abokoura^5^, Taha Yassein^1^, Osama Hegazy^1^, Hany Abdel meguid Shoreem^1^, Hossam Eldeen Mohamed Soliman^1^, Amr Ahmed Aziz^1^, Mohammad Taha^1^, Hazem Mohamed Zakaria^1^, Emad Hamdy Gad^1^, Nahal Kamel Gaballa^2^, Ahmed Nabil Sallam^1^, Islam Ismail Ayoub^1^, Mohamed Eltabbakh^3^, Shimaa Saad Elkholy^4^, Sameh Abokoura^5^, Taha Yassein^1^, Osama Hegazy^1^, Hany Abdel meguid Shoreem^1^, Hossam Eldeen Mohamed Soliman^1^, Amr Ahmed Aziz^1^, Mohammad Taha^1^ actively participated in the preparation, study design, collection of the data and editing of the manuscript. Statistical analysis was done by Hazem Zakaria.

## Registration of research studies

1. Name of the registry: Chinese Clinical Trial Rigestry

2. Unique Identifying number or registration ID: ChiCTR2000030403.

3. Hyperlink to the registration (must be publicly accessible):

http://www.chictr.org.cn/edit.aspx?pid=50331&htm=4

http://www.chictr.org.cn/usercenter.aspx

http://www.chictr.org.cn/edit.aspx?pid=167901&htm=4.

## Guarantor

***Hazem Mohamed Zakaria,*** Department of Hepatopancreaticobiliary & liver transplant surgery, National Liver Institute, Menoufia University, 32511 Shebin El-koom, Menoufia, Egypt. E-mail: hazemzakariaa@yahoo.com, hazem.zakaria@liver.menofia.edu.eg Tel: +2 01019353448, +9660550785685 Fax: +20482234586; Tel.: +20482222740.

## Consent

The research was conducted ethically in accordance with the World Medical Association Declaration of Helsinki. The patients have given their written informed consent on admission and pre-operative to use their prospective data base and files for research work (and as it is a retrospective study on the previous patients data and records so no need for new consents).

## Provenance and peer review

Not commissioned, externally peer reviewed.

## Declaration of competing interest

No conflict of interest.
